# Semiquantitative single-photon-emission computed tomography /computed tomography study to evaluate concomitant ulnar impaction syndrome in patients presenting with triangular fibrocartilage complex tears

**DOI:** 10.1371/journal.pone.0244256

**Published:** 2020-12-23

**Authors:** Yohan Lee, Dongkyu Oh, Jeong Hee Han, Hyun Sik Gong, Won Woo Lee

**Affiliations:** 1 Department of Orthopaedic surgery, Seoul National University Boramae Hospital, Seoul National University College of Medicine, Seoul, Korea; 2 Department of Nuclear Medicine, Seoul National University Bundang Hospital, Seoul National University College of Medicine, Seoul, Korea; 3 Department of Orthopaedic surgery, Seoul National University Bundang Hospital, Seoul National University College of Medicine, Seoul, Korea; IRCCS Polyclinic San Marino Hospital, ITALY

## Abstract

**Introduction:**

Patients presenting with tears of the triangular fibrocartilage complex (TFCC) can have ulnar positive variance, for which the clinical relevance to concomitant ulnar impaction syndrome (UIS) may be unclear. We hypothesized that maximum standardized uptake value (SUVmax), a semiquantitative single-photon-emission computed tomography/computed tomography (SPECT/CT) value, would distinguish between the traumatic TFCC tear and degenerative TFCC tear associated with the UIS. This study aimed to compare SUVmax between patients with TFCC tear only and patients with TFCC tear and UIS.

**Methods:**

A total of 26 patients presenting with TFCC tears on magnetic resonance imaging (MRI) underwent semiquantitative SPECT/CT examinations. The diagnosis of concomitant UIS was made based on positive ulnar impaction tests and MRI findings. We compared the SUVmax between patients with and without concomitant UIS. We also calculated the cutoff value for the diagnosis of UIS using receiver operating characteristic curve analysis.

**Results:**

Of 26 patients, 14 had concomitant UIS, and 12 had TFCC tears only. The SUVmax was significantly higher in patients with concomitant UIS than in those without UIS (p = 0.048). With a SUVmax cutoff value of 4.09 for UIS, sensitivity of 67% and specificity of 82% were obtained.

**Conclusions:**

In the semiquantitative SPECT/CT examinations of patients with TFCC tears, those with concomitant UIS had a higher SUVmax than those without UIS. Semiquantitative SPECT/CT can be helpful in confirming concomitant UIS in patients with TFCC tears.

## Introduction

Ulnar-sided wrist pain is a chief complaint often encountered by musculoskeletal physicians. However, if the history and symptoms are nonspecific, it is difficult to determine the cause of ulnar-sided wrist pain. Triangular fibrocartilage complex (TFCC) tear is one of the frequent causes of ulnar-sided wrist pain. TFCC tears can occur with trauma or degeneration, and degenerative tear was frequently accompanied by ulnar impaction syndrome (UIS) [1]. UIS usually develops in patients with positive ulnar variance, but positive ulnar variance does not necessarily indicate UIS [1]. A traumatic TFCC tear can occur in positive ulnar variance without definite ulnar impaction. If patients presenting with TFCC tears show ulnar positive variance, concomitant UIS diagnosis can be difficult, especially when the type of TFCC tear is unclear and ulnocarpal bone lesions are not shown on magnetic resonance imaging (MRI).

Diagnostic arthroscopy is the gold standard for the diagnosis of ulnar-sided wrist pain [2]. It can directly show the cause of ulnar-sided wrist pain unexplained by MRI but is more invasive and expensive than other imaging tests. Although a hand surgeon can perform surgical treatment right after a definite diagnosis through arthroscopy, patients often refuse to proceed with the operation before being explained with more confidence regarding their diagnosis. For this reason, hand surgeons often require additional tests before diagnostic arthroscopy.

Nuclear medicine can provide additional clues in this situation. Several studies reported that SPECT/CT has diagnostic value for nonspecific wrist pain [3,4]. SPECT/CT can present a clue for bone lesions, which other tests cannot, but it also has several limitations. SPECT/CT images show the qualitative result that requires further interpretation [5]. Traditional SPECT/CT only provides information on the presence or absence of lesions. Even if hot uptake lesions are found, verification of its clinical significance is necessary because the hot uptake may be asymptomatic, representing degenerative changes [5]. The hand surgeon should interpret the SPECT/CT images based on clinical findings. Therefore, even for the same SPECT/CT image, interpretation may differ depending on the physician.

To overcome these limitations, the standardized uptake value (SUV), a semiquantitative parameter, used in positron emission tomography (PET), has been actively studied in SPECT/CT recently [6]. Semiquantitative SPECT/CT provides objective values for a specific disease that qualitative SPECT/CT could not. The SUV represents the image in a semiquantitative format and can aid in excluding bias by interpreters. There are several methods for calculating the SUV, but the maximum SUV (SUVmax) method is the most useful in SPECT/CT for the musculoskeletal system [7,8]. SUVmax is calculated using the strongest uptake value in a volume of interest (VOI).

We hypothesized that SUVmax, a semiquantitative SPECT/CT value, would distinguish between the traumatic TFCC tear and degenerative TFCC tear associated with the UIS. This study aimed to compare SUVmax between patients with TFCC tear only and patients with TFCC tear and UIS.

## Methods

### Patient selection and clinical evaluation

**Fig 1:**
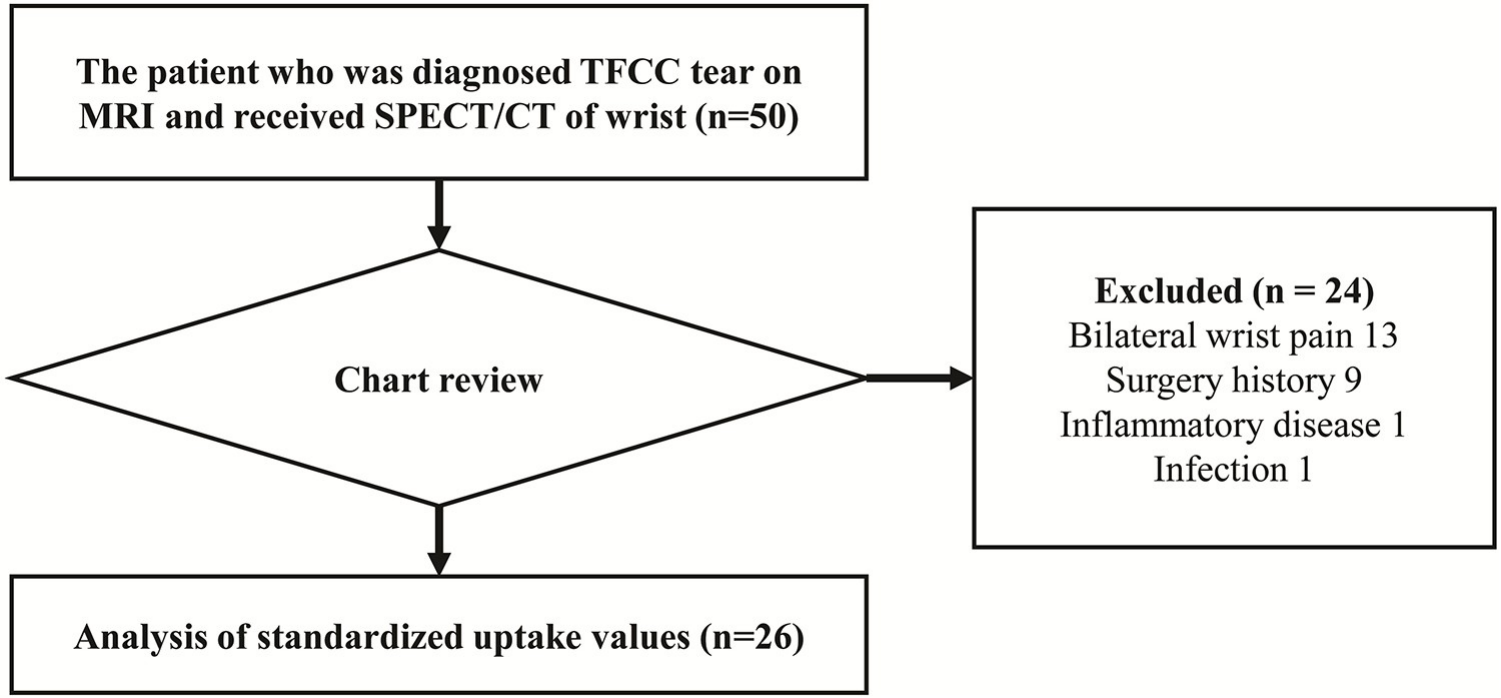
Flow diagram of participants in this study. TFCC, triangular fibrocartilage complex; SPECT/CT, single-photon-emission computed tomography/computed tomography.

We received Institutional Review Board (IRB) approval (B-1910/571-104) for this study and waiver of the requirement to obtain informed consent from the Seoul National University Bundang Hospital. This study retrospectively reviewed the charts between September 2017 and November 2018 for three months starting September 2019. We included all patients who were diagnosed with unilateral TFCC tears on MRI and underwent wrist SPECT/CT at our institution, which is a referral training hospital. The exclusion criteria were bilateral wrist pain, history of hand and wrist surgery, inflammatory arthritis, and wrist infection. Initially, 50 patients were reviewed, and 26 patients were finally analyzed after applying the exclusion criteria (Fig 1).

**Fig 2:**
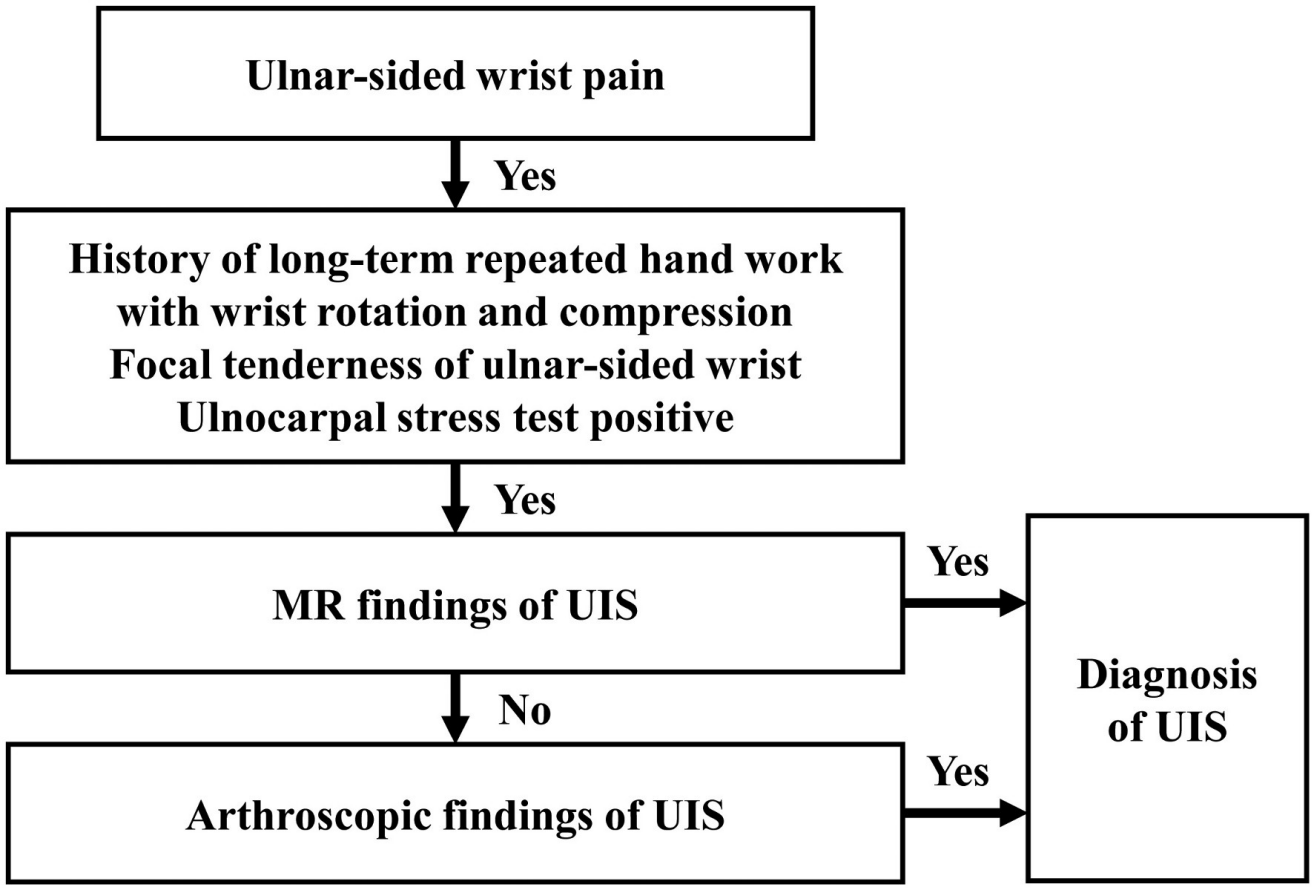
Diagnostic process of ulnar impaction syndrome (UIS). MR, magnetic resonance; UIS, ulnar impaction syndrome.

We diagnosed UIS based on the diagnostic flowchart shown in Fig 2. Plain radiography and MRI were performed when there were clinical signs suggestive of UIS. If both clinical symptoms and imaging studies indicated UIS, diagnosis was confirmed. The MRI findings of UIS included chondromalacia, cysts, or signal changes on the carpal bones or distal ulna. If UIS was clinically suspected but imaging studies were unclear, the diagnosis was made when UIS was detected by arthroscopy. The diagnosis of UIS in arthroscopy was defined as chondromalacia of DRUJ or degenerative tear of TFCC. We did not consider the SUVmax in the diagnosis of UIS.

Two musculoskeletal radiologists evaluated the TFCC tear patterns on the MRIs using the Palmer classification without clinical information [9]. The MRIs were examined using a 3.0-T unit MRI scanner (Achieva and Ingenia; Philips Healthcare, Best, the Netherlands). We measured ulnar variance as the vertical height from the distal ulnar surface of the radius to the distal surface of the ulnar head [10]. Pain level was evaluated using the pain visual analog scale (VAS), an 11-point numerical rating score from 0, “no pain” to 10, “worst pain imaginable” [11].

### Acquisition of SPECT/CT images

Both wrist SPECT/CT were performed to compare the symptomatic and asymptomatic sides. The SPECT/CT was conducted 2–3 h after injection of Tc-99m HDP (dose, 740 MBq) using a dual-head SPECT/CT scanner (NM/CT670, GE Healthcare) equipped with low-energy high-resolution collimator. Patients were placed in prone with the arm in elevation and the elbow joint pronation. Planar images over the hands, wrists, and forearms were first obtained. Then, SPECT images were acquired with the following parameters: energy window peak at 140 KeV (20% window, 126–154 KeV), scatter energy peak at 120 KeV (10% window, 115–125 KeV), step-and-shoot mode, 10 s/step, angle of 3°, total 120 steps (60 steps/detector), body contour option, zoom factor of 1.5, and slice thickness of 2.95 mm. CT parameters were tube voltage of 120 KVp; tube current of 60–210 mA with autoMa function with a noise level of 20; detector collimation of 20 mm (= 16×1.25); helical thickness of 2.5 mm, table speed of 37 mm/s; table feed per rotation of 18.75 mm/rot; tube rotation time of 0.5 s; pitch of 0.938:1; matrix of 512×512; slice thicknesses of 1.25 mm, 0.98 mm, and 0.98 mm (trans-axial, coronal, and sagittal planes, respectively); and no increment and bone plus filtering (ASiR, GE Healthcare).

### Calculation of SUV from the semiquantitative SPECT/CT study

**Fig 3:**
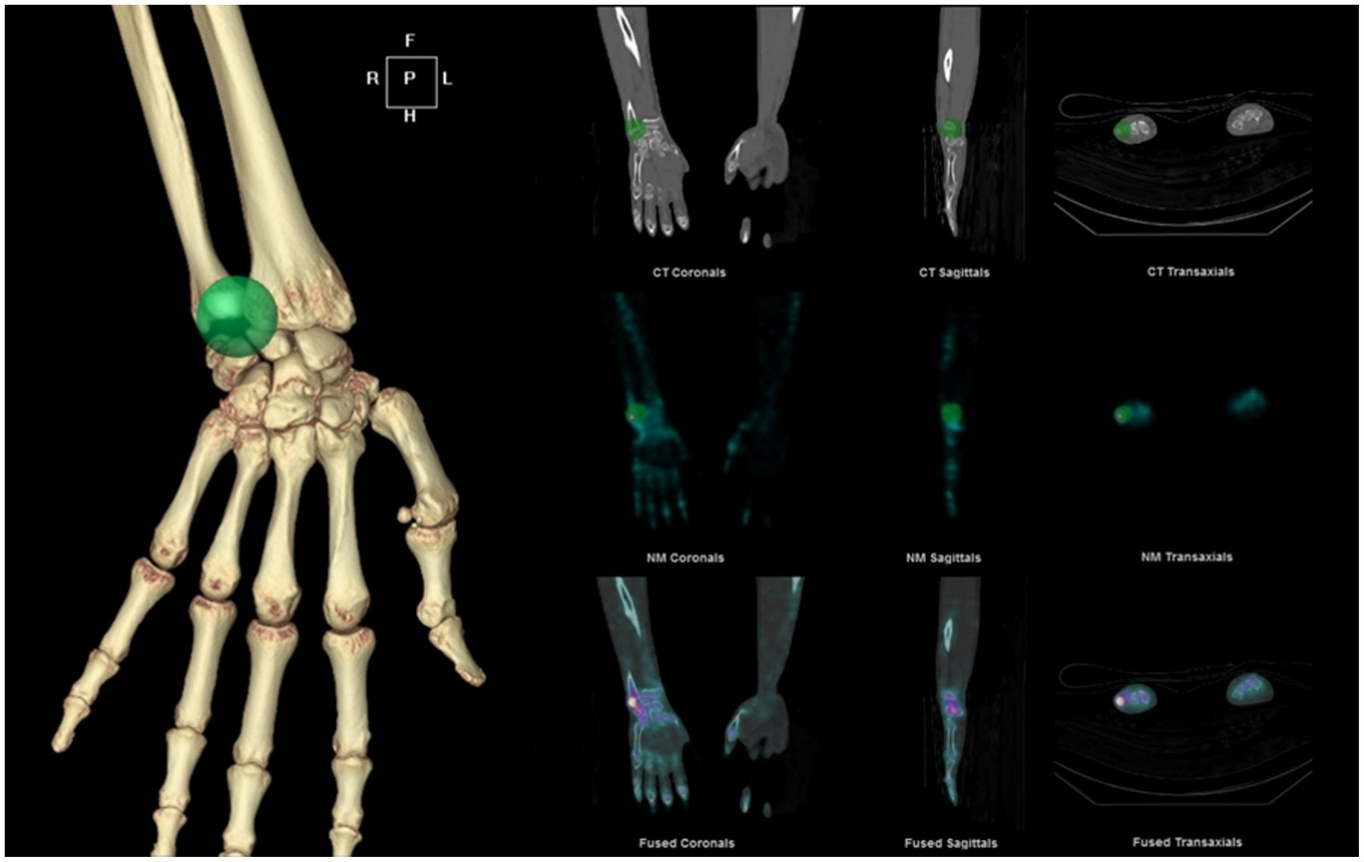
How the volume of interest (VOI) was drawn. A spherical VOI was designed to cover the sigmoid notch of the radius, ulnar fovea, ulnocarpal collateral ligament, and lunate–triquetrum in place in three dimensions on CT. The maximum SUV (SUVmax) of the illustrated VOI was calculated for analysis (SUVmax = 9.07 for this case).

Besides the usual SPECT/CT acquisition process as described above, the following procedures were required to calculate SUV. First, at the time of Tc-99m HDP injection, we recorded the injection time, radioactivity in the syringe, and measurement time (before and after the injection). Second, SPECT images were reconstructed in conjunction with the triad corrections for radioactivity (CT-based attenuation correction, dual-energy window scatter correction, and resolution recovery) into 128×128 matrix during iterative ordered subset expectation maximization (2 iterations and 10 subsets) with a post-reconstruction low-pass filter (Butterworth with frequency 0.48 and order 10) (preparation for Q.Metrix, GE Healthcare). Third, the reconstructed SPECT/CT images were analyzed using a quantitation software (Q.Metrix, GE Healthcare) with an established system sensitivity of 68.4 counts/sec per MBq, which had been obtained from three individual sessions of phantom studies. The method of SUV measurement is presented in Fig 3: a spherical VOI was placed to cover the sigmoid notch of radius, ulnar fovea, ulnocarpal collateral ligament, and lunate–triquetrum. A same-sized VOI was copied and pasted to the contralateral side. The voxel volume for the SUVmax was fixed at 3.2×10^−3^ mL. We calculated the SUVmax as follows [7]:

$$SUVmax=\frac{maximum\;radioactivity\;/\;voxel\;volume}{injected\;radioactivity\;/\;body\;weight}$$

### Statistical analysis

We aimed to compare the SUVmax between the wrist with and without concomitant UIS on the symptomatic side. To verify that the SUVmax obtained from the UIS represents a symptomatic state, we calculated the SUVmax of both the symptomatic and asymptomatic wrists. In reporting descriptive data, the mean and standard deviation were used as continuous variables, and numbers were used as categorical variables. We used the chi-square test for the comparison of categorical variables. Normal and continuous variables were compared through the Student’s t-test. For non-normal variables, the Mann–Whitney U test was used. We did not simultaneously apply the Student’s t-test and Mann–Whitney U test to one variable comparison. The Levene’s test was used for the homogeneity test. The correlation between SUVmax and ulnar variance or pain VAS was analyzed using Pearson’s correlation coefficient. A P-value < 0.05 was defined as statistically significant. We also calculated the cutoff values of UIS using receiver operating characteristic curve analysis. Sample size analysis was performed using G* Power (version 3.1.9.4, Franz Faul, Universität Kiel, Germany). Post hoc power analysis indicated 95% statistical power of the Student’s t-test results in the SUVmax between the wrist with and without concomitant UIS on the symptomatic side.

## Results

### Patient characteristics

The average age of 26 enrolled patients was 37 years (range, 20–63 years), 19 (73%) males, and 14 (54%) right-sided (Table 1). Of the 26 patients, 14 (54%) were diagnosed with concomitant UIS. All patients with UIS showed positive sign in ulnar impaction test. The ulnar variance was statistically different between the patients with concomitant UIS and those with TFCC tears only (3.0 mm vs 0.4 mm, p < 0.001). The distribution of Palmer classifications for the TFCC tears was also different between the groups (p < 0.001). There were no statistical differences in age, sex, side, pain VAS, or symptom duration between the groups. Three patients, who were interpreted as traumatic TFCC tear on MRI, were diagnosed with UIS through arthroscopic findings.

**Table 1:** Patient characteristics.

Variables		Total (n = 26)	TFCC tear + UIS (n = 14)	TFCC tear only (n = 12)	*P*-value
**Age (years)**		37 ± 12	41 ± 13	32 ± 8	0.064
**Male**		19	11	8	0.500
**Right side**		14	9	5	0.249
**Ulnar variance (mm)**		1.8 ± 1.8	3.0 ± 1.6	0.4 ± 1.0	<0.001
**Pain VAS**		6.3 ± 2.0	5.9 ± 1.8	6.8 ± 2.2	0.225
**Symptom duration (months)**		21 ± 22	21 ± 21	20 ± 24	0.920
**Palmer classification**	**1A**	3	2	1	<0.001
	**1B**	6	0	6	
	**1A + 1B**	6	1	5	
	**1B + 2A**	3	3	0	
	**2A**	4	4	0	
	**2B**	2	2	0	
	**2C**	2	2	0	

Data are presented as either mean ± standard deviation or number.

TFCC, triangular fibrocartilage complex; UIS, ulnar impaction syndrome.

### Comparison of SUVmax between patients with and without concomitant UIS

The symptomatic sides showed higher SUVmax than the asymptomatic sides (*p* < 0.001) within each patient. The SUVmax was significantly higher in patients with concomitant UIS than in patients with TFCC tears only (*p* = 0.048, Table 2). The average SUVmax of the wrist with UIS was 4.96, which was higher than that of the symptomatic wrist. There was no significant correlation between SUVmax and pain VAS (r = 0.11, p = 0.586) or ulnar variance (r = -0.27, p = 0.173).

**Table 2:** Comparison of SUVmax.

Variables		SUVmax	*P*-value
**Pain**	**Symptomatic side**	4.07 ± 2.50	<0.001
	**Asymptomatic side**	2.16 ± 0.81	
**Diagnosis**	**TFCC tear + UIS**	4.96 ± 2.81	0.048
	**TFCC tear only**	3.03 ± 1.65	

Data are presented as mean ± standard deviation.

SUVmax, maximum standardized uptake value; TFCC, triangular fibrocartilage complex; UIS, ulnar impaction syndrome.

### Cutoff value of SUVmax for the diagnosis of concomitant UIS

**Fig 4:**
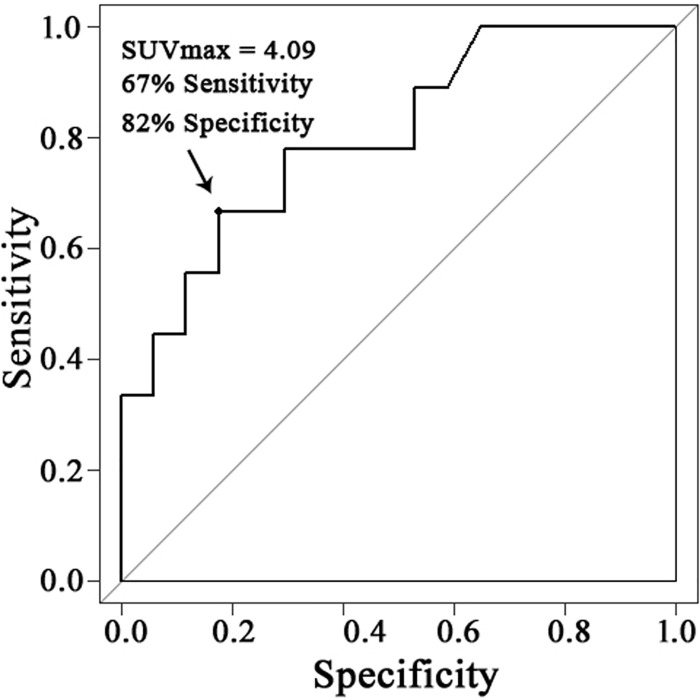
Receiver operating characteristic analyses of the semiquantitative parameters for bone lesions of ulnar-sided wrist pain. Maximum standard uptake values (SUVmax) had an AUC of 0.801 (95% CI, 0.582 to 0.932; p = 0.039). When the SUVmax cutoff value for UIS was 4.09, sensitivity of 67% and specificity of 82% were obtained.

The optimal SUVmax cutoff value to distinguish TFCC tear with UIS from a TFCC tear without UIS was 4.09. With a cutoff SUVmax of 4.09, sensitivity of 67% and specificity of 82% were obtained (Fig 4). The AUC value of the SUVmax for the diagnosis of concomitant UIS was 0.801 (95% CI, 0.582 to 0.932, *p* = 0.039).

## Discussion

In this study, we confirmed that patients with TFCC tear and UIS had higher SUVmax than those with TFCC tear only. The cutoff SUVmax for TFCC tear accompanied by UIS was 4.09. This result suggests that SUVmax can serve as a criterion in identifying UIS accompanying TFCC tears.

The diagnosis of the exact cause of ulnar-sided wrist pain is challenging because various structures are complexly organized in a small area [12]. MRI is commonly used in the diagnosis of TFCC tears. However, we must interpret it with clinical symptoms in mind because the result may be a false positive. In a comparative study of MRI and arthroscopic findings for TFCC tear by Schmauss et al., MRI’s sensitivity was 73–76%, and the specificity was 41–44% [13]. According to a study by Iordache et al., the prevalence of TFCC tear in asymptomatic wrists reached 37.9% [14]. Diagnostic arthroscopy has been suggested as the gold standard if the diagnosis was unclear by MRI [2,15]. However, patients’ reluctance to undergo surgery may hinder an accurate diagnosis.

**Fig 5:**
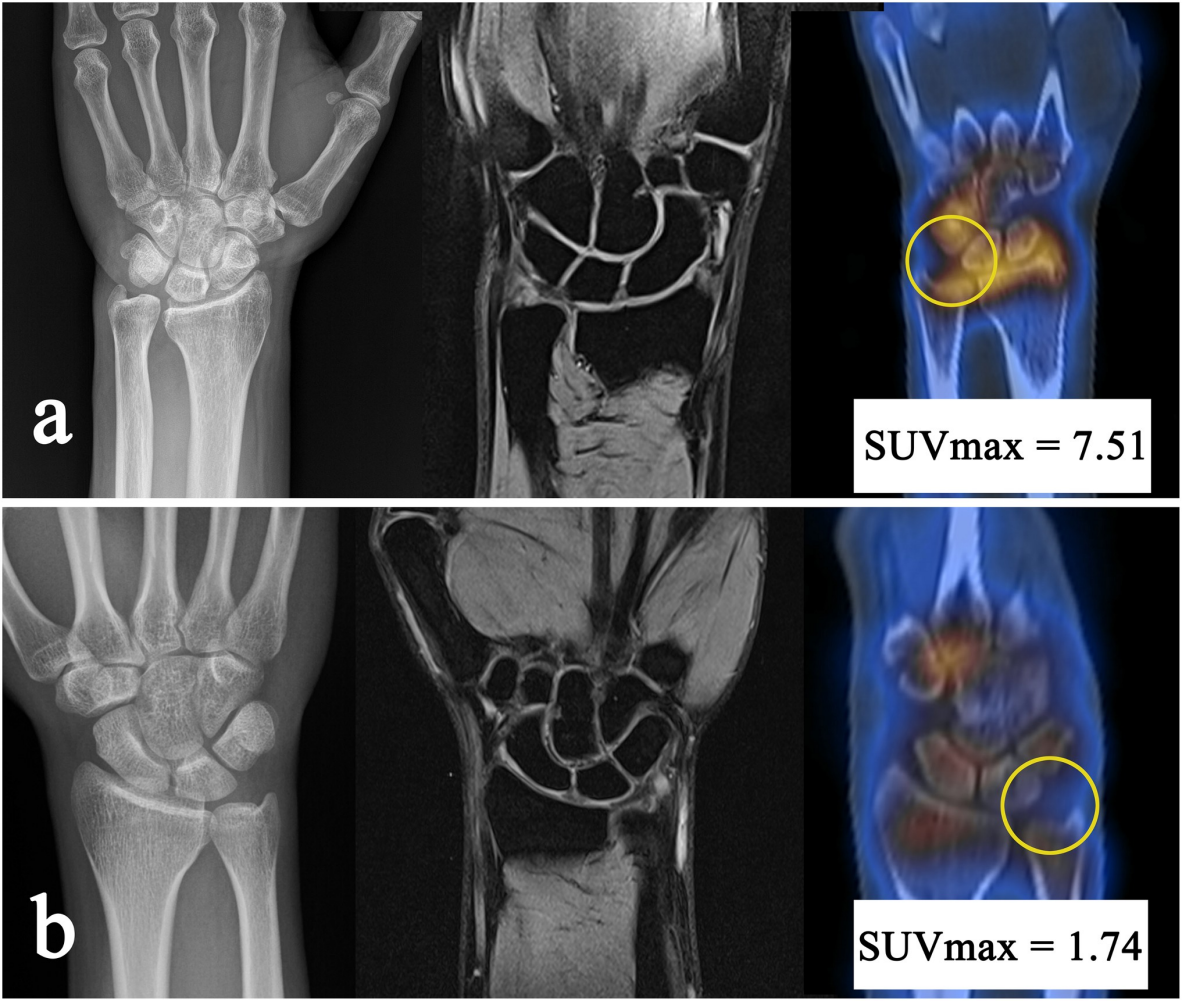
Examples of clinical applications of the maximum standard uptake values (SUVmax). (a) Images of a 47-year-old man. Plain radiography shows a positive ulnar variance. Magnetic resonance imaging (MRI) suggests central and foveal tear of the triangular fibrocartilage complex (TFCC), but there is no evidence of bone bruises or cystic change of the lunate. The semiquantitative SPECT/CT shows a 7.51 SUVmax for the ulnar-sided wrist, indicating active ulnar impaction syndrome (UIS). This patient could be diagnosed with UIS. (b) Images of a 22-year-old man. Plain radiography shows a positive ulnar variance. TFCC of the MRI suggested tear of the ulnar styloid and foveal attachment. There were no typical findings of the UIS between the carpal bones and distal ulna. The semiquantitative SPECT/CT shows mild uptake of the ulnar-side wrist and SUVmax of 1.74 for the ulnar-sided wrist that is lower than the cutoff value of 4.09 for UIS. This patient could be diagnosed with a TFCC tear without UIS.

In this situation, SPECT/CT can be used as an alternative diagnostic tool. Shirley et al. reported that, of 21 patients whose diagnosis was unclear by other tests, 11 patients with SPECT/CT found clear lesions, and 10 of them underwent surgery based on additional findings [3]. In a comparative study of MRI and SPECT/CT on 32 patients with nonspecific wrist pain conducted by Huellner et al., SPECT/CT was reported to be superior to MRI in interobserver agreement and diagnostic accuracy, excluding lesion typification [16]. Strobel et al. concluded that SPECT combined with CT arthrography showed high concordance with MR arthrography in the diagnosis of UIS [17]. Our study advanced one step further from previous studies. We demonstrated that patients with TFCC tear with UIS had higher SUVmax and suggested a diagnostic cutoff SUVmax with sensitivity of 67% and specificity of 82%. Additional information on bone lesions through SUVmax clarifies UIS diagnosis associated with TFCC tears, providing diagnostic confidence before an operation. Fig 5 shows an example of how assessing the SUVmax could help confirm the diagnosis of UIS when bony changes on the MRI are not observed.

**Fig 6:**
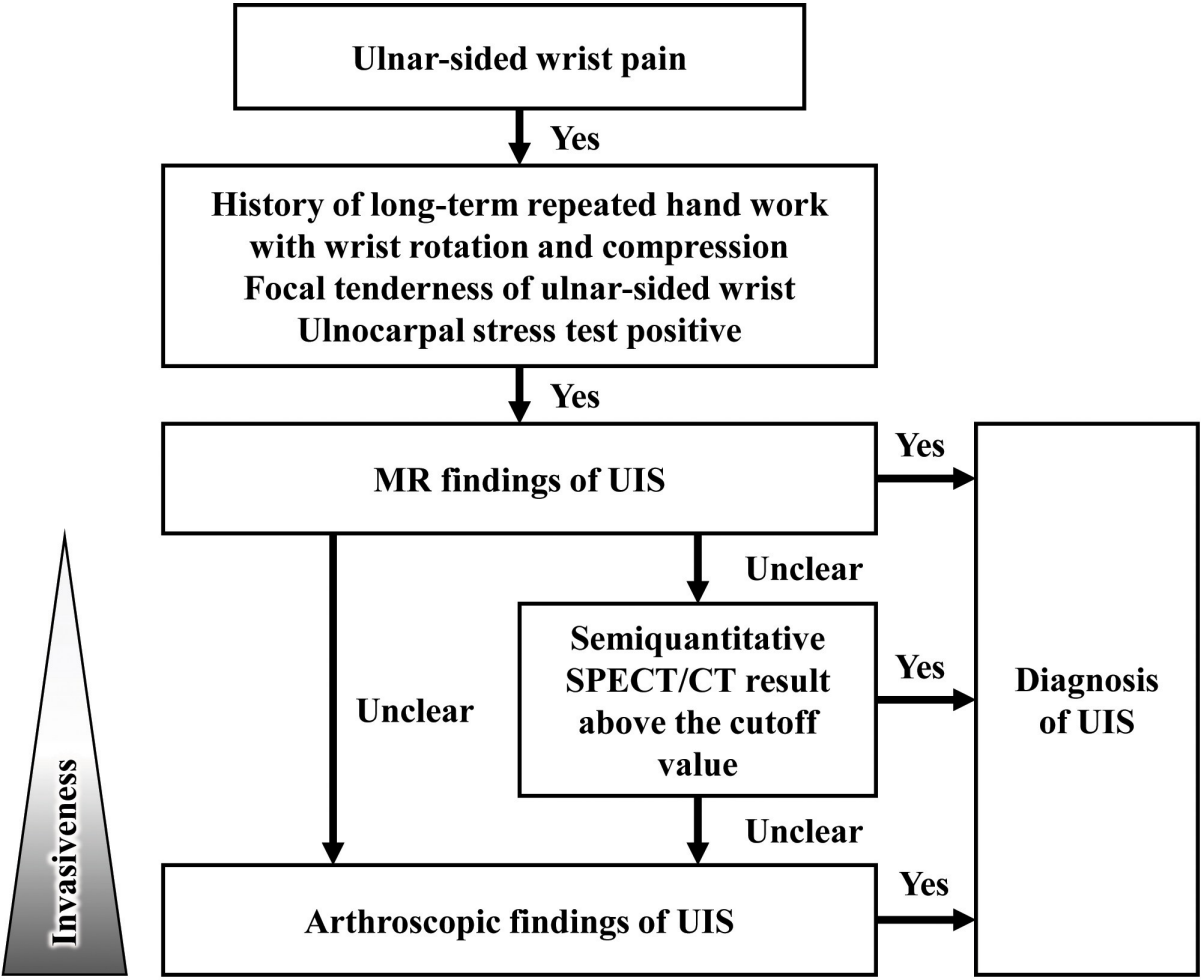
Ulnar-sided wrist pain assessment protocol.

Despite the usefulness of SUVmax, semiquantitative SPECT/CT cannot completely replace MRI and diagnostic arthroscopy. In our study, the sensitivity of SUVmax was 67%, which is insufficient to suggest SPECT/CT prior to MRI. Single-phase quantitative SPECT/CT in this study is focused on a bone lesion, such as UIS, and its narrow range of diagnosis is also a limitation. Radiation exposure of 3–4 mSv per SPECT/CT scan can be a burden, especially for younger patients [18,19]. MRI is still the right choice for second-line investigation after physical examination and plain radiography. When the MR finding is unclear, the surgeon may perform diagnostic arthroscopy, followed by appropriate surgical treatments. However, when a patient desires more confidence in preoperative diagnosis or refuses diagnostic arthroscopy, the quantitative SPECT/CT using SUVmax can be a good third-line investigation (Fig 6).

The evaluation of concomitant UIS in patients with TFCC tears is important because they may need bone surgery in addition to TFCC repair or debridement. Tatebe et al. recommended ulnar shortening osteotomy in the case of positive ulnar variance because mechanical loading to the TFCC can negatively influence healing [20]. In addition, Ruch and Papadonikolakis reported worse TFCC repair outcomes in patients with neutral or positive ulnar variance compared to those with negative variance [21]. However, Reiter et al. showed no statistically significant correlation between ulnar variance and outcome of TFCC repair [22]. This controversy regarding the effect of additional ulnar shortening osteotomy for TFCC tears can result from the difficulty in confirming concomitant UIS in TFCC tears. Further studies using semiquantitative SPECT/CT may reveal the true effect of ulnar-shortening osteotomy in patients with TFCC tears and positive ulnar variance.

Our findings are an extension of results of previous studies on the use of SUVmax for the musculoskeletal system. Suh et al. showed that the SUVmax was the most useful among the quantitative SPECT/CT parameters in temporomandibular joint disorder [7]. The SUVmax was also correlated with radiologic grading and clinical severity in medial compartment osteoarthritis of the knee [23]. Bae et al. reported that high SUVmax were associated with symptoms, surgical treatment as a final decision, and high-risk type of accessory navicular bone [8]. These studies suggest that SUVmax can be a useful tool in evaluating the severity of joint disease.

This study had a few limitations. First, 12 of 26 patients were diagnosed with UIS without arthroscopic confirmation. Although the diagnosis was performed based on clear clinical findings, the diagnosis may be inaccurate when there is no arthroscopic confirmation. The second limitation is the small number of patients. This is a retrospective study of patients who underwent wrist MRI and SPECT/CT in a single institution, and the number of patients is limited. A small number of patients reduced statistical significance and negatively affected the sensitivity and specificity of SUVmax. This is a preliminary study on the validation of semiquantitative SPECT/CT for UIS. For further validation of SUVmax, a prospective study with a larger number of patients is needed. Third, the patients were all referred from primary or secondary institutions, which indicates that the patients had symptoms for more than three months. The long onset may limit the generalizability of our findings. Lastly, our study used a single-phase semiquantitative SPECT/CT that focused on bone lesions only. In a follow-up study, SUVmax can be combined with multi-phase quantitative SPECT/CT to increase the diagnostic value.

## Conclusion

In the semiquantitative SPECT/CT examination of patients with TFCC tears, those with concomitant UIS had higher SUVmax than those without UIS. Semiuantitative SPECT/CT can be helpful in confirming concomitant UIS in patients with TFCC tears.

## Supporting information

S1 Raw data (DOCX)
