# Supplementary material for: Semiquantitative single-photon-emission computed tomography /computed tomography study to evaluate concomitant ulnar impaction syndrome in patients presenting with triangular fibrocartilage complex tears
Source: PLoS One. 2020 Dec 23;15(12):e0244256. doi: 10.1371/journal.pone.0244256 (PMC7757893; doi:10.1371/journal.pone.0244256)
Supplement: S1 Raw data — (DOCX) [file pone.0244256.s001.docx]

Supp 1. Row data

| no. | Age | Sex | UIS | SUV max of symptomatic side | SUV max of asymptomatic side | Palmer | Ulnar variance | Treatment |
| --- | --- | --- | --- | --- | --- | --- | --- | --- |
| 1 | 54 | Female | O | 3.84 | 2.77 | 2C | 5.51 | Conservative treatment |
| 2 | 58 | Male | O | 5.37 | 2.55 | 2B | 2.42 | Conservative treatment |
| 3 | 44 | Female | O | 3.06 | 1.78 | 1A | 4.72 | Ulnar shortening osteotomy+TFCC debridement |
| 4 | 44 | Male | O | 6.16 | 3.31 | 2B | 4.16 | Ulnar shortening osteotomy+TFCC repair |
| 5 | 49 | Female | O | 11.62 | 1.48 | 2A | 1.89 | Conservative treatment |
| 6 | 34 | Male | O | 4.34 | 3.18 | 1A | 2.06 | Ulnar shortening osteotomy+TFCC debridement |
| 7 | 47 | Male | O | 7.51 | 3.06 | 1A+1B | 2.42 | Ulnar shortening osteotomy+TFCC repair |
| 8 | 63 | Male | O | 2.67 | 1.48 | 1B+2A | 5.45 | Ulnar shortening osteotomy+TFCC repair |
| 9 | 20 | Male | O | 4.58 | 2.47 | 1B+2A | 2.98 | Ulnar shortening osteotomy+TFCC repair |
| 10 | 24 | Male | O | 4.16 | 2.15 | 1B+2A | 1.35 | Conservative treatment |
| 11 | 33 | Male | O | 3.1 | 1.06 | 2A | 4.33 | Ulnar shortening osteotomy+TFCC debridement |
| 12 | 44 | Male | O | 2.37 | 1.46 | 2C | 2.59 | Conservative treatment |
| 13 | 29 | Male | O | 1.53 | 1.25 | 2A | 1.08 | Ulnar shortening osteotomy+TFCC debridement |
| 14 | 25 | Male | O | 9.07 | 2.02 | 2A | 0.86 | Ulnar shortening osteotomy+TFCC debridement |
| 15 | 23 | Male | X | 3.8 | 1.9 | 1B | 0 | TFCC repair |
| 16 | 26 | Male | X | 2.45 | 1.35 | 1B | -1.05 | Conservative treatment |
| 17 | 38 | Male | X | 3.85 | 3.76 | 1B | 0.56 | TFCC repair |
| 18 | 39 | Male | X | 7.49 | 3.59 | 1B | 0.27 | TFCC repair |
| 19 | 49 | Female | X | 1.79 | 1.75 | 1A+1B | -1.06 | Conservative treatment |
| 20 | 40 | Female | X | 2.37 | 1.18 | 1A+1B | 0.63 | TFCC repair |
| 21 | 24 | Male | X | 1.76 | 2.06 | 1A+1B | 0.75 | Conservative treatment |
| 22 | 23 | Male | X | 1.57 | 1 | 1B | 0 | TFCC repair |
| 23 | 30 | Male | X | 1.83 | 2.5 | 1A | 2.05 | Conservative treatment |
| 24 | 35 | Male | X | 2.36 | 1.5 | 1A+1B | 0 | Conservative treatment |
| 25 | 31 | Female | X | 3.03 | 2.76 | 1B | 1.4 | Conservative treatment |
| 26 | 29 | Female | X | 4.02 | 2.83 | 1A+1B | 1.69 | Conservative treatment |
